# Supplementary material for: Enhanced Nutritional and Functional Recovery in Femur Fracture Patients Post-Surgery: Preliminary Evidence of Muscle-Targeted Nutritional Support in Real-World Practice
Source: Geriatrics (Basel). 2024 Nov 27;9(6):153. doi: 10.3390/geriatrics9060153 (PMC11675484; doi:10.3390/geriatrics9060153)
Supplement: Supplementary file 1 [file geriatrics-09-00153-s001.zip › geriatrics-3289613-supplementary.pdf]

Supplementary material

Supplementary tables

Supplementary table 1. Exclusion criteria

Supplementary Table 2. Oral nutritional supplementation composition, 100 ml

Supplementary Table 3. Baseline laboratory parameters

Supplementary Table 4. Spearman correlation between changes in nutritional status, functional capacity and muscle strength and baseline characteristics

Supplementary Table 5. Spearman correlation between changes in nutritional status and functional capacity and the change in other outcomes

Supplementary Table 6. Financial support details

Supplementary figures

Supplementary Figure 1. Compliance with nutritional recommendations

Supplementary Figure 2. Level of tolerability with the ONS

Supplementary Figure 3. Level of satisfaction with the ONS

Supplementary table 1. Exclusion criteria

Exclusion criteria
• Declining, unwilling, or intolerant to dietary interventions or ONS despite indication.<br>• Currently receiving enteral nutrition.<br>• Severe cognitive impairment (GDS ≥ 5).<br>• Diabetes not adequately controlled according to the most recent determination (HbA1c ≥ 7%).<br>• Established chronic renal failure according to the most recent determination (GFR < 30ml/min) or with nephrotic syndrome.<br>• Diagnosis of concomitant serious illnesses significantly compromising the nutritional status of the patient, including:<br>• Cancer at any level of the digestive tract (except colon cancer).<br>• Chronic inflammatory diseases at any level of the digestive tract.<br>• Any advanced or end-stage disease as determined by the medical team.
ONS: oral nutritional supplementation, GDS: global deterioration scale, HbA1c: haemoglobin A1C, GFR: glomerular filtration rate

Supplementary Table 2. Oral nutritional supplementation composition, 100 ml

Energy	kcal	151
Fats	kcal/g	5.2
Carbohydrates	g	14.81
Dietary fibre	g	1.4
Proteins	g	10.42
Whey protein	g	9.7
Leucine	g	1.50
Vitamin D	µg	5
Contains other vitamins, minerals, and trace elements		

Supplementary Table 3. Baseline laboratory parameters

Laboratory test results	
HDL (mg/dl) mean (±SD)	47.42 (±13.26)
LDL (mg/dl) mean (±SD)	65.97 (±23.72)
Creatinine (mg/dl) mean (±SD)	1.05 (±0.48)
Folic acid (ng/ml) mean (±SD)	5.10 (±2.25)
Vitamin B12 (ng/ml) mean (±SD)	397.69 (±208.24)

Glucose (mg/dl) mean (±SD)	100.29 (±24.17)
Haemoglobin (g/dl) mean (±SD)	11.03 (±2.12)
Iron (µg/dl) mean (±SD)	49.00 (±41.55)
Ferritin (ng/ml) mean (±SD)	239.81 (±134.43)
Transferrin (mg/dl) mean (±SD)	179.97 (±42.31)
Saturation (%) mean (±SD)	19.35 (±18.22)

HDL: high density lipoprotein; LDL: low density lipoprotein; SD: standard deviation

Supplementary Table 4. Spearman correlation between changes in nutritional status, functional capacity and muscle strength and baseline characteristics

		**Baseline characteristics**						**Days with ONS**
		Age	MNA®-SF score	Barthel Index	Barthel Index score ≥60	Lawton & Brody scale	Muscle strength (kg)	
**Change in MNA®-SF**	Spearman's rho (ρ)	-0.16	-0.05	0.43	0.23	0.06	0.15	0.13
	p	0.38	0.80	0.01	0.21	0.75	0.42	0.47
**Change in Barthel Index**	Spearman's rho (ρ)	-0.10	0.46	0.29	-0.18	-0.11	0.41	0.09
	p	0.58	0.01	0.11	0.33	0.54	0.02	0.64
**Change in muscle strength**	Spearman's rho (ρ)	0.10	0.08	0.29	0.37	0.23	-0.15	-0.18
	p	0.59	0.68	0.10	0.04	0.21	0.41	0.34
**Change in Lawton & Brody scale**	Spearman's rho (ρ)	-0.004	0.13	0.47	0.35	0.23	-0.12	0.48
	p	0.98	0.49	<0.01	0.06	0.21	0.52	<0.01

MNA®-SF: Mini Nutritional Assessment Short Form, ONS: oral nutritional supplementation

Supplementary Table 5. Spearman correlation between changes in nutritional status and functional capacity and the change in other outcomes

		Change between V1 and V2						
		Nutritional status (MNA®-SF)	Barthel Index	Lawton & Brody scale	Muscle strength (kg)	Levels of albumin	Levels of cholesterol	Lymphocyte count
**Change in MNA®-SF**	Spearman's rho (ρ)	-	0.24	0.41	-0.06	0.38	0.45	0.29
	p	-	0.19	0.02	0.76	0.04	0.01	0.11
**Change in Barthel Index**	Spearman's rho (ρ)	0.24	-	0.46	-0.14	0.56	0.38	0.02
	p	0.19	-	0.01	0.45	<0.001	0.04	0.92

MNA®-SF: Mini Nutritional Assessment Short Form

Supplementary Table 6. Financial support details

Aspect	Financial support
Study design	None. There was no financial support or influence from the funding source on the study design.
Data collection	Financial support was provided to facilitate data collection for the project.
Data analysis	None. The funding source provided no financial support and had no influence on the data analysis.
Data interpretation	None. No financial support was provided, and the funding source had no influence on the interpretation of the data.
Writing of the report	Financial support was provided for preparing and writing the report.
Decision to submit the paper	None. The funding source did not influence the decision to submit the paper for publication.
Medical writing	Financial support was provided for writing the manuscript.
Publication fees	Financial support was provided to cover submission and article processing charges.

Supplementary Figure 1. Compliance with nutritional recommendations

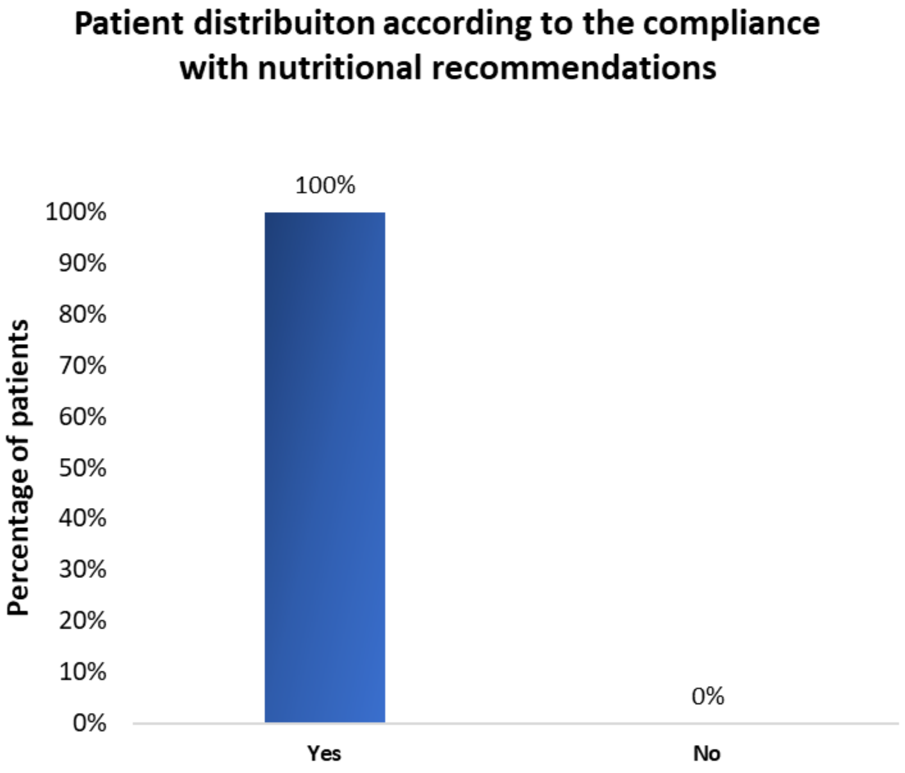

Supplementary Figure 2. Level of tolerability with the ONS

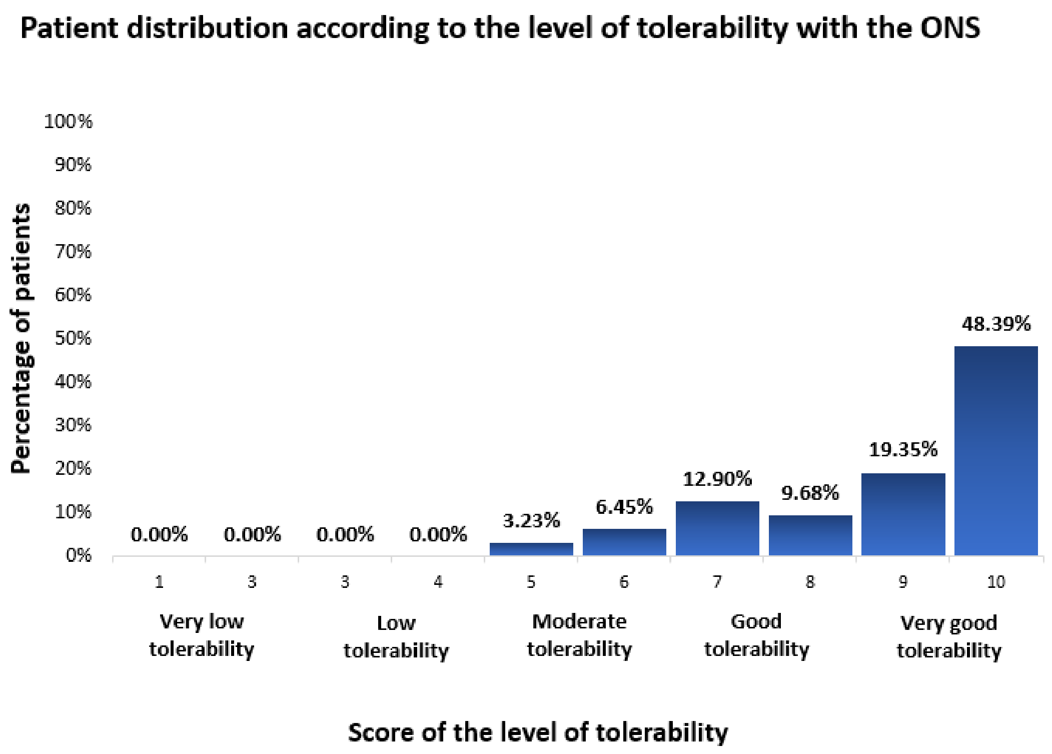

ONS: oral nutritional supplementation

Supplementary Figure 3. Level of satisfaction with the ONS

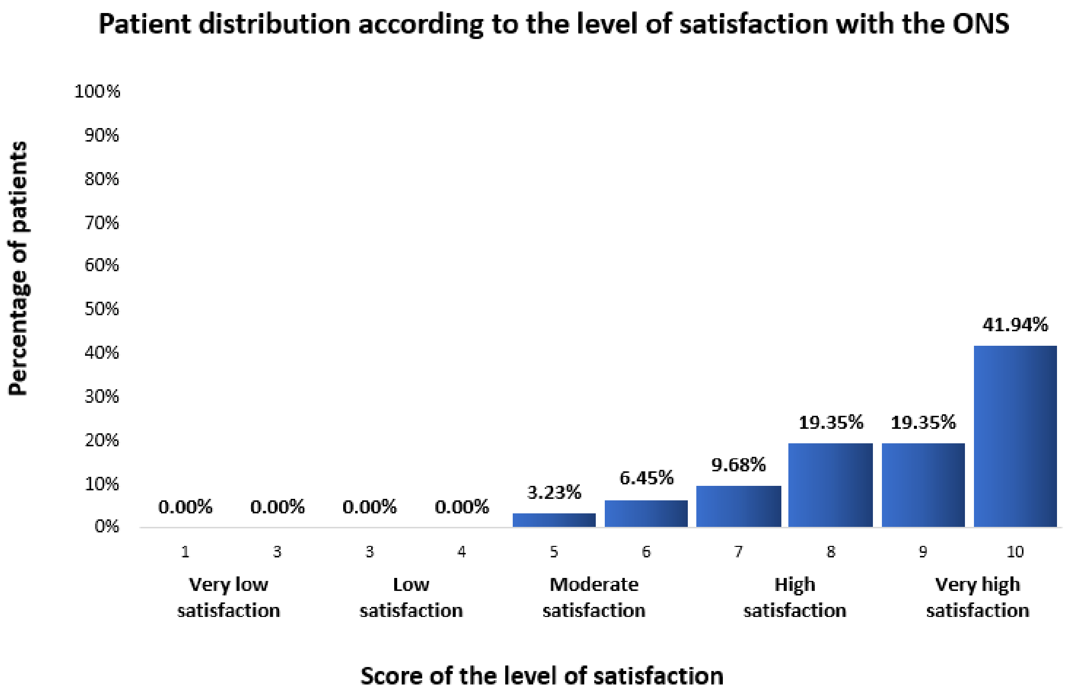

ONS: oral nutritional supplementation
